# Stigma Experienced by Rural Pregnant Women with Substance Use Disorder: A Scoping Review and Qualitative Synthesis

**DOI:** 10.3390/ijerph192215065

**Published:** 2022-11-16

**Authors:** Victoria Bright, Julia Riddle, Jean Kerver

**Affiliations:** 1 Division of Public Health, College of Human Medicine, Michigan State University, Traverse City, MI 49684, USA; 2 Department of Family Medicine, Michigan State University, Traverse City, MI 49684, USA; 3 Department of Epidemiology and Biostatistics, College of Human Medicine, Michigan State University, Traverse City, MI 49684, USA

**Keywords:** rural, pregnancy, substance use disorder, stigma

## Abstract

Identification and recognition of experiences of rural pregnant women with substance use disorder is needed to inform public policy and medical training. This paper reviews and qualitatively synthesizes literature exploring the experiences of this population. Embase, PubMed, and Web of Science were used to identify literature through August 2022 using the search terms, such as pregnancy, substance use or abuse, stigma, and rural. Cited and citing research were also considered. Exclusion criteria included articles that failed to consider rural pregnant women’s perspectives on stigma experienced, included potential confounds, occurred outside of the United States or Canada, and were published before January 2000. Nine articles met the inclusion criteria. Data were synthesized by the listed authors and assessed for common themes. A review of the articles revealed three common themes: stigma occurs in community settings, stigma occurs in healthcare settings, and comprehensive care is important to ensure appropriate support to this population. Stigma as a barrier seems to improve when women have strong social support and access to comprehensive care networks. Addressing this stigma through programs, such as peer social guidance and comprehensive health centers, may provide appropriate support to pregnant, rural women with SUD to further navigate their health needs.

## 1. Introduction

Substance use disorder (SUD) remains an issue that impacts many different populations, including pregnant women. According to reports from the Substance Abuse and Mental Health Services Administration (SAMHSA), past month use rates for pregnant women in 2019 were 10% for alcohol, 10% for tobacco, and 6% for illicit drug use [1]. Of illicit drugs, 5% of pregnant women reported marijuana use, and <1% reported opioid use in the past month. However, SAMHSA reports national trends, and substance use across communities does not necessarily match national averages. For instance, a different study found that 3% of the women hospitalized for childbirth in Maine matched the diagnostic criteria for opioid use disorder [2].

Further, pregnant women who use opioids are at risk of enduring significant stigmatization by the public. To address this topic, Racine et al. (2015) explored the effect of different addiction models, biological versus moral, on stigmatization experienced by pregnant women with SUD [3]. They found that people seem to perceive non-pregnant people who use drugs as having a biologically derived addiction, which shifts the blame away from a person’s character. Pregnant women, however, were viewed as having a biological and moral basis for addiction. That is, others simultaneously view them as having a biological “fault” driving them to use drugs and a moral “fault” leading to the inability to avoid using drugs [3]. Such stigmas penetrate social settings, creating a significant barrier for women to achieve a sense of well-being.

While SUD and the associated stigma experienced by pregnant women likely occur in both rural and urban communities, there are nuances in how SUD may impact these communities. Gabrielson et al. (2020) explored the rates of opioid use disorder and comorbid conditions in Maine’s rural and urban women during hospitalization for labor and delivery [2]. Researchers found that the subset of rural women delivering was more likely to meet the diagnostic criteria of opioid use disorder at delivery than their urban counterparts. Further, rural pregnant women with SUD have unique social factors mediating how they interact with their communities. These women face difficulty finding and accessing treatment centers for opioids and other substances [4]. Additionally, their access to care may be further complicated by a lack of anonymity within a rural community. Rural communities are unique settings where the boundaries between community and medical settings are blurred [5]. That is, a doctor is a neighbor, and vice versa.

The prevalence of SUD in rural environments and the unique social characteristics that encompass the communities may further complexify seeking care for rural pregnant women with SUD. Specifically, the stigma these women may experience due to the blurred boundaries of rural communities may limit the accessibility to care. The stigma within the community may exacerbate the treatment inaccessibility and complicate the women’s perception of self, making them less likely to seek care. This literature review aims to address these topics by establishing how rural pregnant women who use illicit drugs experience stigmatization in social and medical settings and explore possible mechanisms to lessen this issue.

## 2. Materials and Methods

### 2.1. Study Design

A qualitative synthesis, as defined by Seers, was conducted to identify and synthesize existing literature regarding the stigma that rural pregnant women with SUD experience [6]. To maintain a structured review method, despite this being a scoping review, we modeled our review methods after the Preferred Reporting Items for Systematic Reviews [7].

The literature review focused on populations in the United States and Canada. Including literature only from the United States and Canada was done in an effort to limit possible confounds across different governments and cultures. Specific outcomes explored in the literature include evaluating the experiences of the defined population during the prenatal and peripartum periods. Additionally, we considered how the experiences impact pregnancy and SUD care. The protocol of this literature review was not registered.

### 2.2. Study Selection

**Figure 1:**
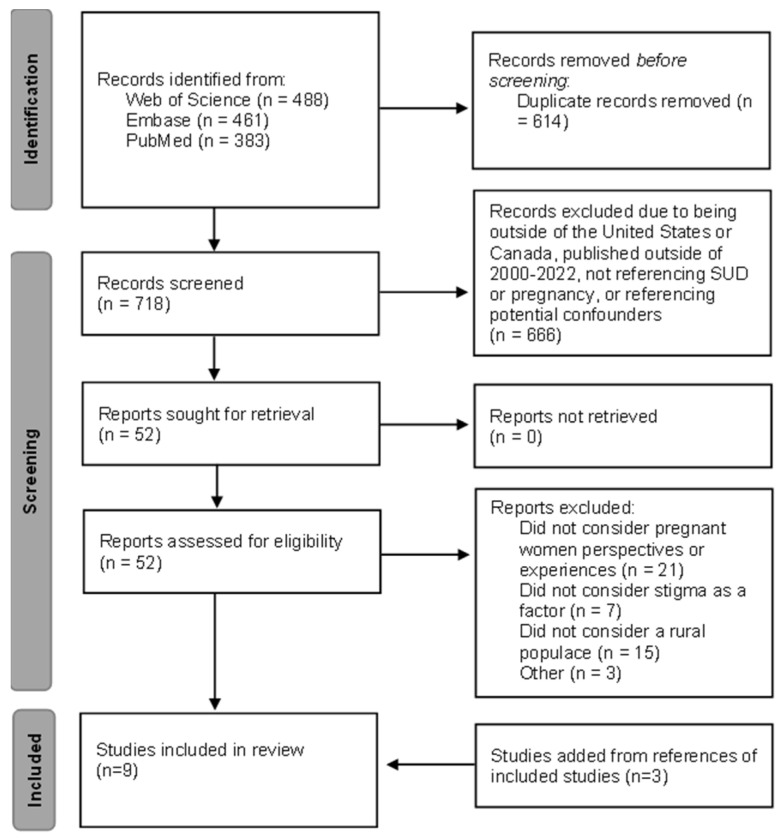
Stepwise literature review. Flow chart adapted from the Preferred Reporting Items for Systematic Reviews [7].

Information databases used to conduct the literature search included PubMed, Web of Science, and Embase. The literature search identified relevant literature published through 7 August 2022 using the following keywords: pregnancy, substance abuse, stigma, and rural. Variations of the keywords (i.e., substance abuse and substance use disorder) and variable word combinations were employed. Language such as abuse was used to identify older articles and those that include severe misuse without clearly defining SUD. Additional identification resulted from snowball sampling by reviewing citations in each selected article and searching for research that cited the selected articles. Figure 1 provides further details regarding the search strategy.

### 2.3. Criteria for Inclusion

The primary objective of this literature review was to establish how rural pregnant women with SUD experienced stigma in the community and medical settings. Specifically, we included primary research studies published between 1 January 2000, and 7 August 2022, focusing on the perspective and experiences of rural pregnant women with SUD. The article language was restricted to English. From the articles collected using the above keywords, we excluded research that included variables that may confound stigma experiences (i.e., HIV status, mental health conditions, familial violence); was conducted outside of the United States or Canada; and failed to reference rural populace, stigmatization, or pregnant women’s perspectives. Literature reviews were also excluded.

We did not exclude papers with postpartum participants if the research questions reflected the experience while pregnant. We also did not exclude papers that included polysubstance use due to the commonality of such a practice and the small sample size with exclusion. Finally, we did not exclude papers that failed to clearly define SUD but instead indicated severe substance use through syntax such as “addiction” and “substance abuse”.

### 2.4. Data Extraction

Researchers reviewed and evaluated the articles for the above inclusion and exclusion criteria. Titles were initially reviewed to rule out literature beyond the scope of this paper. The remaining papers’ abstracts were reviewed, and we completed an in-depth review of the last articles collected following this screening procedure.

The remaining 52 articles were read and categorized further. A numerical description of the articles included and excluded is presented in Figure 1. Of note, the three articles excluded as “other” presented in Figure 1 were studies missed during the prior screening steps. The references of the six articles that met the inclusion and exclusion criteria were considered as well. We then categorized the included results based on apparent themes. Results were compared between researchers, and discrepancies were resolved through further discussion and analysis.

## 3. Results

Following the literature search, nine articles met all inclusion criteria and were included in the literature review. Table 1 provides details of the final selected articles. Three themes arose from the literature, and they are described further below.

**Table 1:** Characteristics of the selected literature reviewed. Substance use disorder—SUD. NICU—neonatal intensive care unit—NICU. Years old—y.o. Opioid use disorder—OUD. Neonatal abstinence syndrome—NAS. Obstetrics and Gynecology—OB/GYN.

First Author (Publication Year)	Study Aim	Study Setting	Participants	Data Collection	Analysis
Blaire [8]	Characterize experiences of perinatal persons with SUD	University of Kentucky, NICU	11 postpartum women (≥18 y.o.), with perinatal OUD and children requiring NAS observation/treatment	Semi-structured interviews	Sandelowaski’s qualitative description method
Burgess [9]	Learn about stigma affected seeking and maintaining treatment for opioid use disorder	MaineGeneral Health	2 groups of postpartum women who sought treatment while pregnant (≥18 y.o.), 8 groups of non-postpartum men and women (≥18 y.o.)	Standardized interviews, recorded focus groups	Summative content analysis, conventional content analysis
Jackson [10]	Examine how barriers in seeking substance use treatment differ between urban and rural pregnant women	University of Kentucky Medical Center	Pregnant women (≥18 y.o.) undergoing inpatient detoxification	Face-to-face interviews, medical record review	Thematic analysis of interviews, quantitative analysis of socio-demographic variables
Jackson [4]	Examine barriers to entering substance use treatment experienced by rural, pregnant women	University of Kentucky Chandler Medical Center	Pregnant women (≥18 y.o.) undergoing inpatient detoxification	Face-to-face interviews, medical record review	Thematic analysis of interviews, quantitative analysis of socio-demographic variables
Jessup [11]	Examine extrinsic barriers to substance abuse treatment for “pregnant drug-dependent women”	Northern California	Perinatal women (24 weeks pregnant-1 year postpartum, (≥18 y.o.) undergoing residential treatment	Semi-structured interviews	Qualitative life history analysis
Kramlich [12]	Explore the perception of rural women with SUD during their pregnancy and infants’ hospitalization; explore how the perceptions impacted their ability to bond with their baby	Northeast United State	Perinatal women (pregnant or recently birth, (≥18 y.o.) identified as having opioid use disorder	Semi-structured interviews	Framework analysis and thematic analysis
Ostrach [13]	Explore ambivalence about medication-assisted treatment for OUD in the context of societal stigma	Central Appalachia	Pregnant women receiving medication-assisted treatment and prenatal care at an OB/GYN office	Observation and semi-structured interviews	Thematic analysis
Paterno [14]	Describe experiences of addiction in pregnancy, recovery, and a mentor/peer support role	Massachusetts	Peer mentors with a history of substance use during pregnancy	Digital storytelling workshop and semi-structured interviews	Analysis using intertextual transcripts and thematic analysis (ground theory approach)
Roberts [15]	Explore how drug use and factors associated with drug use became barriers to care	Northern California	Perinatal women (pregnant-2 years postpartum, (≥18 y.o.) undergoing residential treatment	Semi-structured interviews and focus groups	Thematic analysis

### 3.1. Stigma within the Community

Multiple researchers in various locales establish the importance of social support in seeking care and the barriers set by stigma within the community. Jackson and Shannon suggested that acceptability and accessibility were significant barriers for rural pregnant women with SUD seeking care [10]. The authors also presented that these barriers existed in urban populations; however, they also suggested that a lack of anonymity in rural areas may exacerbate these barriers [4]. Lack of anonymity reinforcing the feelings of stigma among pregnant women with SUD is further supported by reports of paranoia while filling out “Medi-Cal forms and everything” in public places due to the fear that others will learn about substance use [15].

Jackson and Shannon report that 15% of the rural sample identified stigma as one component of their acceptability barrier [4]. These women further qualified this feeling by describing a fear of being judged if family and friends discovered their status of being pregnant with SUD, as they thought that others would label them as a “fallen woman” [4]. Additionally, Paterno et al. (2019) reported how a woman felt that her actions, such as “nodding off,” were thought to be secondary substance use by others, and this perceived scrutinization limited the motivation to access resources to treat their SUD and pregnancy [14].

There may be a geographical variance in the role of social support. Studies in Kentucky identify social support as an accessibility barrier (i.e., lack of assistance in childcare), but Northern California studies identify it as an acceptability barrier [10,15]. Roberts and Pies suggest that isolation from family and friends perpetuates the barriers to seeking care [15]. This isolation is multifactorial—due to fear of disclosing their status as a pregnant woman with SUD, lack of familial support for other reasons, and loss of peers who also use drugs. Such isolation further enhances the stigma experienced by pregnant women with SUD [15].

Like the population in Kentucky, a population of Northern California pregnant women suggested that different people felt the right to discuss substance use with them. However, pregnant women often did not find these conversations helpful due to the lack of insight regarding the difficulties of seeking care [15]. Rather, having others tell them what they need to do while concurrently juggling the complexities of achieving care as pregnant women with SUD led to excess stress. Roberts and Pies further argue that better informing the population about the effects of drug use while pregnant is necessary to improve such interactions [15]. Public health messages discussing the harm of drug use during pregnancy motivate the conversations. However, the public viewpoints and underlying misunderstanding of outcomes and processes to seek care can exacerbate the community’s stigma, further limiting the acceptability of women seeking care [15,16].

The stigmatization experienced does not appear limited to seeking treatment, and it may continue as the women actively participate in treatment for SUD [13]. Ostrach and Leiner argue that this influence extends into pharmaceutical treatment, as family and friends perceive opioid maintenance therapy as a temporary solution, which may diminish women’s management efforts [13]. As such, pregnant women with SUD face a paradoxical dilemma—stigma prevents them from seeking care, and stigma about care received decreases treatment motivation. These findings support that intrinsic motivation to seek care positively correlates with acceptability barriers [10]. That is, women want to seek care but are severely limited by the social stigma experienced.

Despite the potential barriers to care, social support can also motivate seeking care. Ostrach and Leiner discuss the importance of having a “fortress of support” [13]. Many participants indicated that they would have likely relapsed due to stigma in the community and healthcare settings without support from others. Roberts and Pies report similar findings [15]. Women in both studies reported variable efforts, such as a boyfriend motivating them through the journey, neighbors aiding with insurance enrollment, and familial reminders of the irrelevance of others’ thoughts. Paterno et al. (2019) report that one participant endorsed that positive social support “seems to be the key and across the board for everybody... in recovery” [14].

### 3.2. Stigma within Healthcare Settings

Pregnant women with SUD are aware of the stigma in healthcare settings [13]. Approximately 50% of the study participants experience stigma in hospitals during encounters including, but not limited to, labor and delivery [9]. This stigma persists while seeking SUD and prenatal care [9,13].

Ostrach and Leiner considered the barriers to seeking SUD treatment among pregnant women [13]. For instance, one woman had difficulty finding a provider willing to treat her SUD, and a preference for detoxification and hesitancy towards medication therapy further complicated this process. Providers did not support her decision to complete detoxification, even though it is an acceptable—although not preferred—option for pregnant women with SUD [13]. With the help of a provider, the woman finally found a detoxification center willing to help her. Similarly, Kramlich et al. (2018) present one woman with SUD whose long-term provider informed her that care would be discontinued if the woman became pregnant again [12]. Despite the unwillingness to continue seeing the woman, the provider ensured to refer the patient to another physician. In both cases, barriers limited access to care, but providers aimed to connect the patients with care. However, finding new healthcare professionals may worsen the fear experienced and enhance their reluctance to seek general and SUD-related health care [9].

Pregnant women with SUD report an awareness regarding stigma in healthcare settings, and they note that this contributes to anxiety regarding interactions with healthcare and social services [13]. For instance, Ostrach and Leiner report that most participants report having “that one nurse” during their hospitalization in the labor and delivery unit; that is, the women report healthcare workers who openly criticized their medication-assisted SUD treatment and treated them differently than other patients [13]. Blaire et al. (2021) presented multiple women who similarly interacted with healthcare providers that stigmatized them and did not treat them as “people” [8]. These examples were supported by a woman who shared how a nurse suggested that she should not be nervous about blood collection as she used intravenous drugs, an assumption based on a puncture wound left by a previous employee [8]. Further, comments about “ruining” veins were commonly reported [8].

Similarly, Burgess et al. (2021) report that one postpartum group experienced the most stigma while interacting with nurses [9]. Such interactions involved nurses treating the women as if they were drug-seeking or interacting with them in a way that made the women “feel stupid.” Feelings of stigma do not only arise during nursing interactions, as Kramlich et al. (2018) had a participant report physician refusal of a cesarean section because they did not want to give a “junkie” the anesthetics [12]. Women in Massachusetts also felt that staff disregarded their wishes because they were a “junkie mom” by ignoring their parenting preferences, such as not using a pacifier [14]. The reports parallel how some women endorsed that “right things” still feel “wrong” [13].

Not all interactions with healthcare providers obviously contribute to this perception of stigma among pregnant women, as Jessup et al. (2003) report microaggressions against them [11]. Women report that providers use terms like “dirty test results” when discussing their pregnancy’s potential outcomes [11]. Further, the women indicated that these actions, in addition to the custody threats and outcome predictions, affirmed the stigma experienced in such settings. These women also reported that such interactions demotivated them and created a sense of ambivalence toward seeking care. A sense of anxiety and paranoia experienced by pregnant women with SUD seeking treatment worsens the perception of stigmatization [11,13]. Such occurrences do not always appear as specific interactions. Some women feel that doctors do not listen and account for their concerns, contributing to feelings of lack of care [15].

These justifications fail to acknowledge the stigma experienced and their efforts to avoid such interactions, such as traveling to receive care [12]. However, it is crucial to recognize that the stigma observed in healthcare settings encourages behaviors among pregnant women that reinforce physicians’ distrust. Kramlich et al. (2018) describe how women report intentional dishonesty if they feel stigmatized and recognize mistreatment in healthcare settings [12]. This leads to false reports and gaps in care. Further, physicians’ interactions and continuous dishonesty create a feeling of internal stigmatization embodied by guilt, shame, and embarrassment [12].

### 3.3. Importance of Comprehensive Care

Pregnant women with SUD are intrinsically motivated to seek prenatal and SUD care [10]. However, these stigmatizing interactions limit women’s desire to seek care. These experiences lead to inadequate care that affects current and future pregnancies [11]. However, actions as simple as assisting with rides or offering food vouchers can positively impact women [8]. As such, there is a widespread call for more comprehensive, women-centered care for this patient population [4,12].

There appears to be a need for providing comprehensive care that focuses on the various systems these women interact with, such as legal, medical, and social services [4]. Kramlich et al. (2018) report that women who participated in comprehensive care programs report more accessible care [12]. They attributed this to having a single physician and various resources in one location. Roberts and Pies further suggest that this care needs to involve extensive education services to ensure that the women become self-advocates and can explore the system independently [15].

Additionally, the literature demonstrates the importance of developing a supportive and understanding network. Comprehensive centers often incorporate social services, and the participants emphasize the importance of developing personal connections and trusting relationships during treatment [4]. A “fortress of support” is a strong motivator for women undergoing care, and the presence or absence of such care may dramatically alter the treatment outcome [13].

## 4. Discussion

The stigma that pregnant women with SUD experience infiltrate communities in social and medical settings. While such stigma does not seem specific to rural populations, unique considerations complicate the effects of stigma on prenatal and SUD treatment, such as accessibility to treatment, lack of anonymity, and acceptability of treatment options available.

Previous efforts have tried to eliminate barriers to care among this population, such as improving access to treatment centers in rural environments. The stigmatization experienced by these women may thwart these efforts. As such, addressing the stigma seems to be an appropriate avenue to increase care during this period of self-motivation. Multiple articles cited in this literature review emphasized the importance of peer support during treatment engagement, as indicated by the positive impact of social relationships and comprehensive treatment centers. Exploring different options for facilitating community formation may provide insight and opportunities to decrease the stigma recognized by this population. For instance, a recent study investigates how peer mentors help pregnant women with SUD through various means, such as acting as an advocate in healthcare settings and sharing their own experiences as examples [14]. Efforts like this guided social support may be especially beneficial for rural areas with limited resources. Further, programs such as Opioid Health Homes encourage peer recovery mentorship, which provides women with appropriate social support while decreasing the complexity of the healthcare system. Similar programs are being considered in Australia as well [17].

However, it is crucial to remain vigilant, as the stigma appears ingrained in social and healthcare settings. Logan et al. (2003) present that most small-town inhabitants in Kentucky fail to identify maternal alcohol use during pregnancy as a leading cause of poor mental development in infants [16]. Instead, they identify prenatal “crack/cocaine” as the leading cause of delayed development. The sample also mistakenly thought infants were “born drunk” [16]. Logan et al. (2003) also report that some physicians believe that women will not be honest about their substance use [16]. This research group further states that physicians justify the lack of care as access and availability issues, the unwillingness of pregnant women to seek treatment, and fear of breach of confidentiality in rural settings. The misconceptions within the community and healthcare settings perpetuate the stigma experienced by this population.

As such, interventions to lessen the stigma experienced during pregnancy may be essential to promoting SUD treatment. For instance, some research indicates that the motivation to seek care decreases among rural parenting women with SUD, yet these women also face less stigma while seeking care [18]. Specifically, Ali et al. (2022) report that 12% of parenting women recognize a need for SUD treatment [18]. This differs from the 24% of pregnant women who seek SUD treatment [10]. This apparent discrepancy between pregnant and parenting women further iterates the need to eliminate remaining barriers to treatment for women with SUD, as the internal motivation for treatment is higher during pregnancy.

The stigmatization this population experiences seems to extend beyond the North American borders. Research reveals that women in Australia who use various substances, most commonly heroin, indicate self, societal, and healthcare provider stigma all act as a barrier to seeking care [17]. Both groups of women report similar experiences, including having health care providers “stick their nose up” at them or self-isolating from friends and family due to guilt and “feeling different” [4,8,17]. Women in the United Kingdom share similar sentiments, as these women also report stigma in community settings [19]. Recognition of the role of stigmatization in these women seeking care and possible plans to address it may have widespread implications.

The primary limitation of this literature review is the scarcity of research regarding the stigma experienced by this population. In addition, there is limited data investigating how stigma in various subgroups differs. For instance, Burgess et al. (2021) present various settings where women may experience stigma, but the research did not explore how the interactions differed between pregnant and non-pregnant individuals [9]. That is, it is unclear if the data presented is unique to pregnant women. Additionally, limited subpopulation analysis occurred, including variations based on race, religion, or socioeconomic status. The literature seems to over-generalize the experience of stigma in women being treated for SUD, and possible nuances in experiences and interventions may be obscured. Additionally, while this literature review only included studies completed in the United States and Canada, there is further literature regarding the experience of women with SUD in other countries. As such, further research is needed to specify more examples of stigmatizing behavior across groups of people undergoing or seeking SUD treatment. This data would allow for SUD treatment policy changes that further promote care engagement.

## 5. Conclusions

The stigma that rural pregnant women with SUD experience are present in the community and medical settings. Stigmatization, decreased accessibility and acceptability, and lack of anonymity limit this population’s ability to seek SUD and obstetric care. Despite these barriers, pregnancy provides a unique opportunity to interact with these women. Creating comprehensive care paradigms may further promote involvement with prenatal and SUD care. This idea coincides with the rising popularity of centralized care management programs.

## Data Availability

A copy of the literature review results can be obtained by requesting a list from the corresponding author.
